# Hemodynamics analysis of the serial stenotic coronary arteries

**DOI:** 10.1186/s12938-017-0413-0

**Published:** 2017-11-09

**Authors:** Xin Liu, Changnong Peng, Yufa Xia, Zhifan Gao, Pengcheng Xu, Xiaoqing Wang, Zhanchao Xian, Youbing Yin, Liqun Jiao, Defeng Wang, Lin Shi, Wenhua Huang, Xin Liu, Heye Zhang

**Affiliations:** 1 Department of Anatomy, Guangdong Provincial Key Laboratory of Medical Biomechanics, School of Basic Medical Science, Southern Medical University, 1023-1063 Shatai South Road, Baiyun, Guangzhou, 510515 Guangdong China; 2 Department of Cardiology, Shenzhen Sun Yat-Sen Cardiovascular Hospital, Shenzhen, 518055 China; 3 Shenzhen Institutes of Advanced Technology, Chinese Academy of Sciences, 1068 Xueyuan Ave., Xili University Town, Nanshan, Shenzhen, 518055 Guangdong China; 4 Shenzhen College of Advanced Technology, University of Chinese Academy of Sciences, Shenzhen, 518055 China; 5 Shenzhen Keya Medical Technology, Shenzhen, China; 6 Xuanwu Hospital, Capital University of Medical Sciences, Beijing, China; 7 Department of Imaging and Interventional Radiology, Prince Of Wales Hospital, The Chinese University of Hong Kong, Hong Kong, China

**Keywords:** Coronary artery, Multiple stenoses, Computer tomography based fractional flow reserve, Computational fluid dynamics, Geometric factors

## Abstract

Coronary arterial stenoses, particularly serial stenoses in a single branch, are responsible for complex hemodynamic properties of the coronary arterial trees, and the uncertain prognosis of invasive intervention. Critical information of the blood flow redistribution in the stenotic arterial segments is required for the adequate treatment planning. Therefore, in this study, an image based non-invasive functional assessment is performed to investigate the hemodynamic significances of serial stenoses. Twenty patient-specific coronary arterial trees with different combinations of stenoses were reconstructed from the computer tomography angiography for the evaluation of the hemodynamics. Our results showed that the computed FFR based on CTA images (FFRCT) pullback curves with wall shear stress (WSS) distribution could provide more effectively examine the physiological significance of the locations of the segmental narrowing and the curvature of the coronary arterial segments. The paper thus provides the diagnostic efficacy of FFRCT pullback curve for noninvasive quantification of the hemodynamics of stenotic coronary arteries with serial lesions, compared to the gold standard invasive FFR, to provide a reliable physiological assessment of significant amount of coronary artery stenosis. Further, we were also able to demonstrate the potential of carrying out virtual revascularization, to enable more precise PCI procedures and improve their outcomes.

**Electronic supplementary material:**

The online version of this article (doi:10.1186/s12938-017-0413-0) contains supplementary material, which is available to authorized users.

## Background

As the cardiovascular diseases (CVD) has become more prevalent in China, the lack of routine screening and the cost of the screening procedure had increased the population of these disease. Especially, atherosclerotic stenosis caused ischemic event is responsible for 17% of the population and the mortality rate rose to above 40% according to the statistical report in 2013 [1, 2]. The significant ischemic events occurred at different phases of the progression of the stenoses due to the individual variations that serial stenoses in single vessel are commonly found [1]. The serial stenoses was accounted for 39.5% of major adverse clinical outcomes in the patients suffer from myocardial infarction and the recurrent acute coronary syndromes [1, 2]. Fractional flow reserve had been proved reliable on functional assessment of the coronary stenosis [3, 4]. Clinical reports showed that FFR guided percutaneous coronary intervention had reduced the cost of procedures and improved the clinical outcomes [4–6]. However, clinical practices showed conflict findings that the effect of the serial stenosis to the true FFR value of the culprit stenoses is uncertain [7–11]. Although it has been showed that it is safe to repeat invasive FFR in stable patients [11], the invasive nature of FFR and the hemodynamic interference between serial stenoses are still challenge for multiple measurements in practice [12, 13]. Therefore, a noninvasive method is required for further understanding the relationship between anatomies and hemodynamics.

The image-based computational simulation has been used as a noninvasive method for hemodynamic analyses of the stenotic arteries [14, 15]. The accuracy of the CTA-based FFR (FFRCT) had been validated suitable for noninvasive functional assessment that improved efficiency of screening patients with suspected stable coronary artery disease and significantly reduced usage of invasive coronary angiography with non-obstructive disease [14, 16, 17]. The study of flow distribution analysis in representative stenotic arterial segments with various shapes of the lesions suggested that curve arteries are more susceptible to the occurrence of atherosclerosis [18]. Distribution of WSS is also found correlated to the progression of atherosclerosis [19]. But comprehensive understanding of the influence of complex lesion distribution in a single branch of the stenotic coronary artery on hemodynamics is still limited [20, 21].

In the present study, we aimed to evaluate the anatomic parameters for assessing the serial stenoses based on the hemodynamics analysis. The patient-specific coronary arteries with serial stenoses were reconstructed from the computed tomography angiography (CTA) images and the hemodynamic simulations were performed. The anatomic and hemodynamic parameters were collected for further evaluations.

## Methods

### Patient data and 3D reconstructions

The cohort of 45 patients who underwent CTA with suspected ischemic related stenosis were performed with FFR measurement [22]. Criteria were made to exclude the patients with: total occlusion of the coronary arteries; previous myocardial infarction; presented acute symptoms in the previous 60 days; had arrhythmias; had previously undergone coronary artery bypass graft surgery or percutaneous coronary intervention. Twenty patients met the criteria that their image data were retrospectively selected through the approved IRB. 3D anatomical data from a 256 multi-slice CT detector (Siemens definition, Erlangen, Germany) with 0.6 mm slice interval were reconstructed. For enabling accurate analysis of hemodynamics distribution, the preserved reconstructed models extended from the aorta root to all the visible branches of left coronary and right coronary arteries. Details of the coronary geometries were determined from the images, with limited modification to the integrity of the structures. FFR was performed following the clinical practice guideline [23], hyperemia was induced by using an infusion of adenosine (140 μg/kg/min) via the femoral vein [24], and the pullback FFR data were recorded from the immediate downstream of the distal stenosis to the ostium of the coronary. FFR was then calculated as the ratio between mean distal pressure (mPd) and mean aortic pressure (mPa) (Eq. 1) [25].

1 $$ {\text{FFR}} = \frac{\text{mPd}}{\text{mPa}} , $$

All procedures followed the guidelines of clinical practices [23, 26].

### CFD configuration

The blood flow was assumed 3 dimensional, incompressible and pulsatile. The vessel wall was assumed rigid and no-slip boundary. Newtonian fluid was assumed, and the blood viscosity and density were taken to be constant at 0.0035 Pa s and 1060 kg/m^3^ for all simulations [27]. The flow momentum and the mass conservation were carried out by Navier–Stokes governing equations:

2 $$ \rho \left( {\frac{\text{du}}{\text{dt}} + {\text{u}} \cdot \nabla {\text{u}}} \right) = - \nabla {\text{p}} + \mu \nabla^{2} {\text{u}} + {\text{f,}} $$

3 $$ - \nabla \cdot {\text{u}} = 0, $$

where ρ is the density of blood, u is the velocity field, p is the pressure, μ is the viscosity, and f is the body force taken to be zero. The period of simulation time was 8 s, which contains the 8 cycles of variations with a time step size equal to 0.001 s.

The lump parameter model (LPM) was implemented to the outflow boundaries [28] and the LPM parameters were calculated following the flow distribution principle [28]. The inflow boundary was implemented by means of a healthy physiological pressure waveform. The flow distribution into the coronary artery was assumed to be equal to 4% of the cardiac output [29], which provided the baseline reference to each primary branch of the coronary artery; the flow to each daughter branch was then calculated on the basis of the morphology data measured manually from the patient-specific geometries [30]. The resistance values of the LPM were computed according to the Poiseuille law based on the morphology of the vessel and the mean flow rate to the branches. These parameters included coronary arterial resistance and coronary arterial microcirculation resistance, while the coronary impedance spectrum was applied from the literature data [31, 32]. Following the literature procedure, the capacitance values were applied on the basis of the previous study accordingly [28].

The WSS was considered as a contribution factor to improve the assessment of the anatomy-related significant hemodynamics [33]. The WSS is defined as:

4 $$ {\text{WSS}} = \frac{1}{\text{T}}\mathop \int \limits_{0}^{\text{T}} \left| {\mu \frac{{\partial {\text{V}}_{\text{t}} }}{{\partial {\text{n}}_{\text{t}} }}} \right|{\text{dt                                          }} $$

where μ is blood viscosity, n_t_ is velocity vector near wall perpendicular to surface and n is distance to the wall surface, T is pulsatile period, dt is the time derivative of the local shear stress. In addition, the time averaged WSS (TAWSS) [33] for one cardiac cycle was calculated to include the range of WSS.

The variation of the radius in the stenotic vessel was taken as a new indicator for the evaluation of the hemodynamics variation. The center line of the arterial geometries were automatically established, the sampling interval was taken to be 0.2 mm to preserve the detail of the geometric variations. The value of radius was calculated by averaging over the cross-sectional areas at each interval. The mean distal pressure was calculated from the simulations at each sampled point along the centerline of the vessel the mean aortic pressure was calculated at the ostium of the left and right coronaries. The non-invasive FFR (FFRCT) along the vessel was then calculated following Eq. 1.

## Results

### Geometric characteristics of the patients’ coronary arteries

**Fig. 1:**
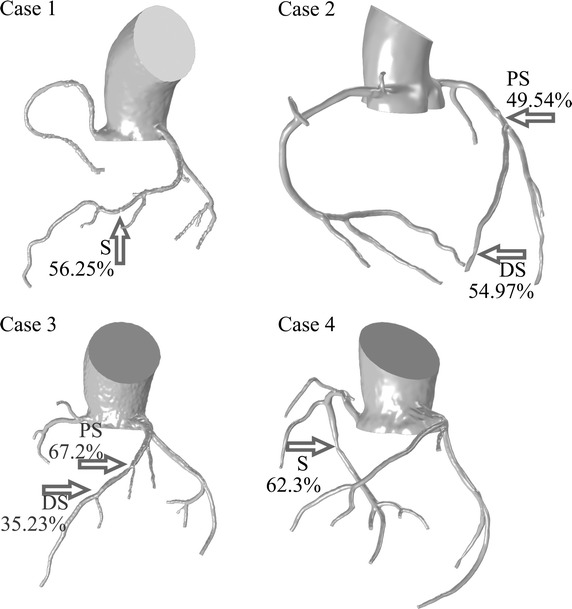
Patient specific coronary arteries with stenosis: significant stenoses are indicated by arrows, and the severities of the stenoses were determined by the structural assessment based on the cross-sectional area of the lumen. The distribution of the isolated stenosis varies between cases, and serial stenosis were found in case 2 and case 3. *S* stenosis; *PS* proximal stenosis; *DS* distal stenosis. For case 1, the stenosis was in the middle of the left anterior descending coronary artery of length 3.28 mm; for case 4, the stenosis was in the middle of the right coronary artery of length 8.34 mm. In case 2, the stenosis was of lengths 3.98 and 2.32 mm distributed at the bifurcation of the LAD and distal daughter branch; in case 3 the stenosis was of lengths 4.84 and 2.11 mm, distributed at the adjacent bifurcation in the LAD. The reconstruction based calculated stenosis percentage areas of the four cases were 56.25% in case 1, 49.54 and 54.97% for the proximal and distal stenosis in case 2, 67.2 and 35.23% for the proximal and distal stenosis in case 3, and 62.3% in the middle of RCA in case 4

The 20 patient-specific coronary geometries were reconstructed. Among all, the 4 typical geometries were taken as examples as illustrated in Fig. 1. Isolated stenosis was found in case 1 and case 4 with significant difference in the length of the lesions. For case 1, the stenosis was in the middle of the left anterior descending coronary artery of length 3.28 mm; for case 4, the stenosis was in the middle of the right coronary artery of length 8.34 mm. On the other hand, serial stenoses were found in (i) case 2 of lengths 3.98 and 2.32 mm distributed at the bifurcation of the LAD and distal daughter branch, and (ii) case 3 of length 4.84 and 2.11 mm distributed at the adjacent bifurcation in the LAD. The severity of the stenosis at the locations of measurement was then calculated from the reconstructions; the stenosis percentage based on cross sectional area reduction of the four cases were 56.25% in case 1, 49.54 and 54.97% for the proximal and distal stenosis in case 2, 67.2 and 35.23% for the proximal and distal stenosis in case 3, and 62.3% in the middle of RCA in case 4.

### Validation of the calculation

The hemodynamic simulations were performed in the 20 patient-specific coronary arterial geometries to investigate the performance of FFRCT in the functional assessment of complex stenosis. Validation of the calculation was made by comparing the calculated FFR at the location similar to the measurements, wherein the invasive FFRs were taken downstream to the stenosis in all cases. The mean ± SD of the error between FFRCT and FFR was − 0.023 ± 0.015. Taking the FFR < 0.8 as indicator of ischemic-related stenosis, the true positive and true negative values of FFRCT was 11/12 (91%) and 8/8 (100%), respectively. FFRCT values in the four example cases were 0.84, 0.67, 0.74 and 0.81, respectively. The corresponding FFR values were 0.83, 0.68, 0.79 and 0.88, respectively. The error between the FFRCT and the FFR in the four cases were 1.2, − 1.4, 6.3, and − 3.4%, the mean standard deviation of the error was − 0.0067 ± 0.042. As can be noted, good agreement was achieved between the calculations and the measurements.

### Pressure distributions in the patient-specific coronary arteries

**Fig. 2:**
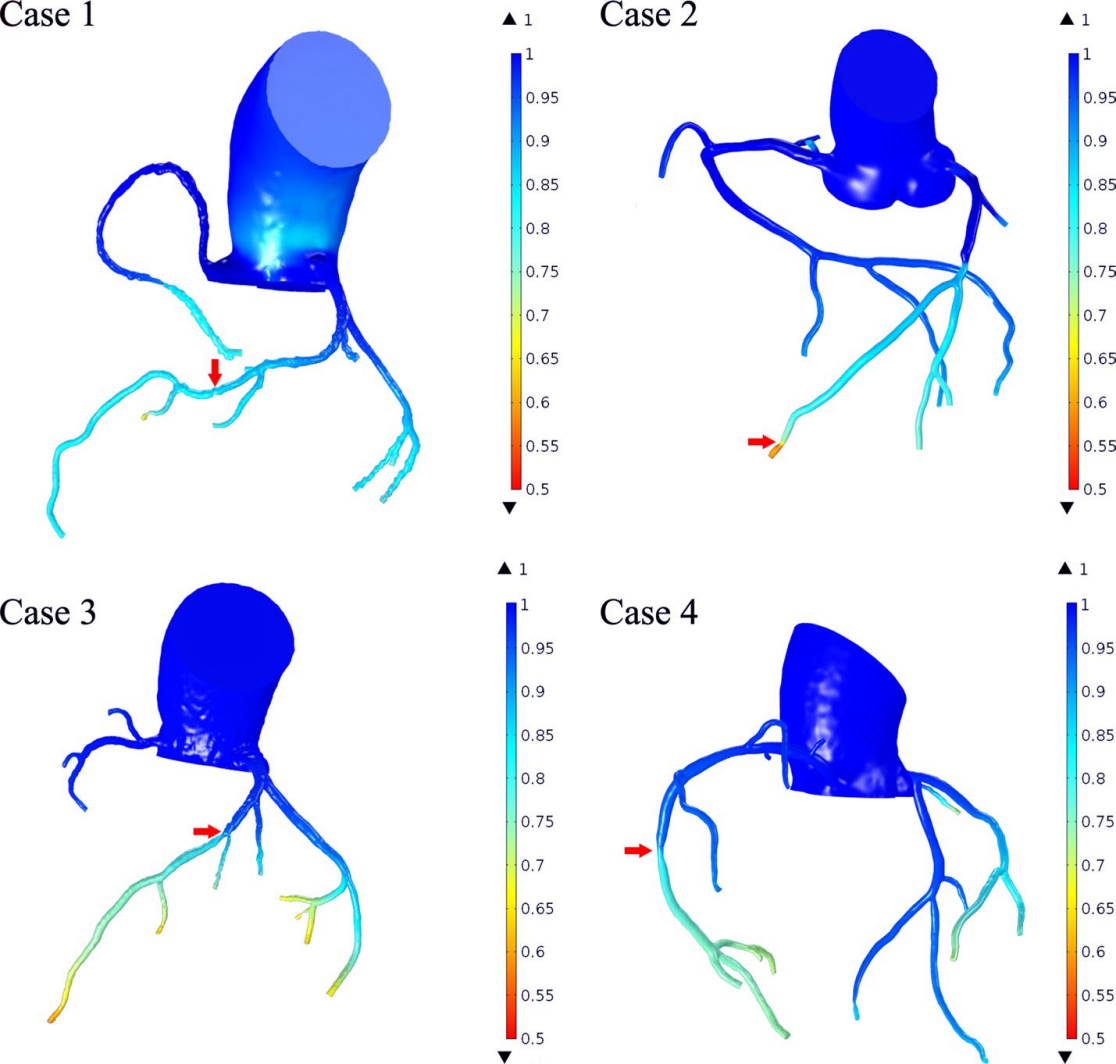
FFRCT gradients of the four cases are illustrated. The calculated FFR values in the four cases were 0.84, 0.67, 0.74 and 0.81. Considerable drop in the FFRCT gradient was found to be located at the significant stenosis (indicated by the arrows), except for case 1

The time average pressure distribution in one cycle for the all cases were calculated (the four typical cases were taken as examples as illustrated in Fig. 2). The pressure drop was not significant at the bifurcation in the normal segments; comparing the left coronary arteries and right coronary arteries, the appearance was similar. Significant blood pressure variations were concentrated at the culprit stenosis, and the pressure drop was negatively proportional to the severity of the stenosis (as indicated by the arrows in Fig. 2).

### Correlation between the pullback curve and the coronary geometries

**Fig. 3:**
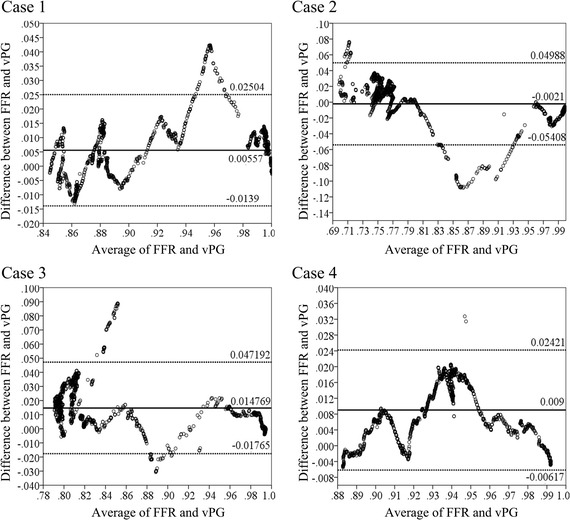
The Bland–Altman agreement test showed good agreement between calculations and measurements. The calculations showed slight underestimation in cases 1, 2 and 4, and slight overestimation in case 2

The pressure values along the target vessels were extracted following the center-line of the vessels, and sampled at uniform distance to form the pullback curves. The distance between sampling points was determined to be half of the length of significant stenosis in the vessel. The locations of the beginning of the pullback curves were determined according to the patient-specific DSA images. The sampling procedure was also performed on the FFR pullback curve in each case, for evaluation of the accuracy of the calculations. The spearmen test showed good agreements to the overall pullback curves in the all cases (mean ± SD was 0.84 ± 0.102 with p value < 0.01, patient-wise). Particularly, for the 4 example cases, the correlation factor was 0.951, 0.923, 0.809, and 0.969, respectively, with p < 0.01, as indicated by Fig. 3). On the other hand, slight overestimation was found for the calculation in case 2 (with mean difference ± SD was − 0.0021 ± 0.02652) and underestimations were found for the remaining cases (with mean difference ± SD of 0.00993 ± 0.00557, 0.01477 ± 0.01654, and 0.009 ± 0.00775 for case 1, case 3 and case 4, respectively) according to the Bland–Altman test (Fig. 3).

**Fig. 4:**
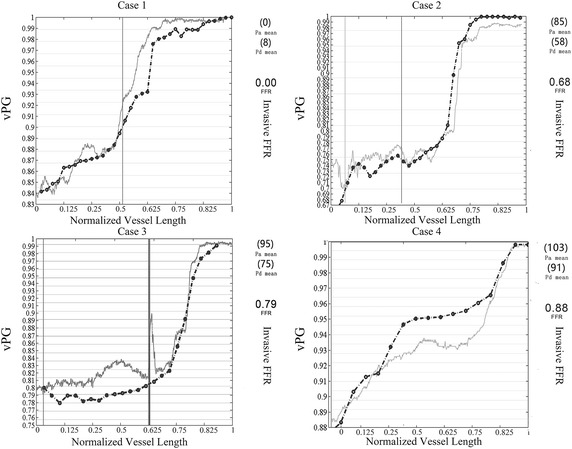
The FFRCT pullback curves were overlapped on the FFR gradient measurements. The x-axis represents the distance from the ostium of the coronary to the stenosis, and distance is normalized. The features of the FFRCT pullback curves (solid line with dots) are similar to that of the FFR gradient (solid lines). The FFRCT pullback curves are less fluctuate compare to the FFR measurements. In both FFRCT and FFR gradians, the pressure recovers from the immediate upstream of the stenosis and the pressure recovery rate decreases between serial stenoses. On the other hand, the FFRCT pullback curves are less fluctuate compare to the FFR gradients. Deviations are found along the stenotic vessel, but the average of deviation is less than ± 0.015. The maximum deviation was over 0.9, however, the mean standard deviation of FFRCT values at the significant stenosis was − 0.0067 ± 0.042

The pattern and the values of the calculated pullback curves are evaluated by overlapping to the invasive FFR pullback curves from the measurements for the example cases (Fig. 4). The solid line represents the FFR and the dashed line with circle marks represents the FFRCT. Some variations in the patterns of the pullback curves were found among the cases. For instance, in case 1, the FFR value represents the pressure distribution in the stenotic left coronary artery; there appears to be a sudden increase from below 0.85 to above 0.88 at the normalized length of 0.25 from the distal beginning, which is an indication of the pressure variation between the immediate downstream and the immediate upstream of the distal stenosis. Within the same segment, the calculated pullback curve is less fluctuating compared to the measurement curve. Following the fast pressure recovery to 0.98 at the normalized length of 0.625, a plateau is observed along the measurement curve (FFR = 0.93), which indicates the pressure variation to the proximal stenosis. A delay is observed in the calculated curve. The calculated underestimation is mainly distributed proximal to the proximal stenosis.

In case 2, a gradual increase is found from the distal end to normalized length of 0.625, the FFR/FFRCT increased from 0.68/0.67 to 0.79/0.79, respectively. According to the DSA images, the distal end located at the downstream of the distal stenosis in the left anterior descending branch of the left coronary artery, and the proximal stenosis was located at the proximal bifurcation of the left coronary artery. The slow recovery of the pressure distribution coincides with the segment between two significant stenoses.

For case 3, slow pressure recovery is also found between the two stenoses in the left anterior descending branch of the left coronary artery. The measurement shows that FFR increased from 0.79 to 0.83 from the distal end to the normalized length of 0.75; the pullback curve could reproduce the pressure level at the two significant stenoses. However, the rate of the pressure recovery from the calculation diverged from the measurement during this segment. The measurement FFR curve fluctuated above 0.8, with a wide pulse between the normalized length of 0.25 and 0.625, followed by a significant short pulse to 0.9 before the normalized length of 0.75; the corresponding geometric characteristics were the proximal stenosis distal to the nearby bifurcation. On the other hand, the calculation presented a pressure drop at the distal stenosis at the bifurcation, followed by a slow pressure recovery until the proximal stenosis.

For case 4, the FFR value recovered from 0.88 to 0.92 over the distal stenosis, which is 0.91 for the calculation at the normalized length of 0.2. Following a plateau along the normalized length of 0.2 to 0.8, a recognizable overestimation is found in the calculation compared to the measurement (an average 3% error comparing FFRCT to FFR along the pullback curves). The corresponding segment begins from the significant stenosis at the mid-right coronary artery to the proximal.

**Fig. 5:**
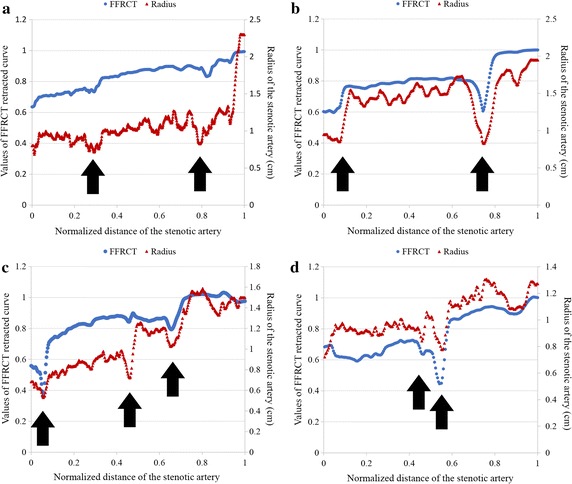
Four cases with serial stenoses were showed as examples here that the radiuses (marked with triangles) of the stenotic arteries were overlapped to the FFRCT (marked with solid circles). The valley of the radius curves showed the distribution of serial stenoses (as indicated with arrows). Plateau of FFRCT were found between valleys of the radius curve along the vessel (as showed in **a**, **b** and **c**). However, when the stenoses became close enough, the plateau became a delay of FFRCT recovery at the immediate downstream of the distal stenosis (as showed in **d**)

A significant drop of FFRCT was found in the cases with single stenosis (case 4 in Fig. 4), on the contrary, a plateau was found in serial stenosis (case 2 and 3 in Fig. 4). Therefore, 14 cases were identified with serial stenosis and grouped that the correlation between radius and FFRCT were evaluated. Spearman test showed that mean ± SD per patient over the 14 cases was 0.9 ± 0.35, p < 0.01). Four cases with serial stenoses were showed as examples here that the radiuses of the stenotic arteries were overlapped to the FFRCT (Fig. 5). FFRCT pullback curves showed plateau between serial stenoses (as showed in a, b and c in Fig. 5). However, as the distance became small between stenoses, the plateau was replaced by a delay of pressure recovery instead (as showed in d in Fig. 5). The correlation between variation rate of the radius and the FFRCT was also tested that the mean ± SD of spearman correlation per cases was 0.56 ± 0.08, p < 0.0001.

### Correlation of wall shear stress to the pressure variations

**Fig. 6:**
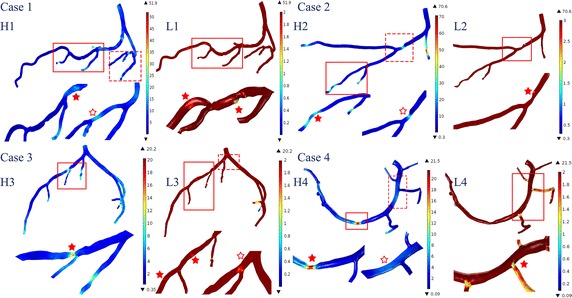
The TAWSS in the stenotic arteries showed variations due to the distribution of the stenoses and the geometric characteristics of the arterial tree. H1, H2, H3, H4 and the L1, L2, L3, L4 indicate the distribution of high and low TAWSS in each case, respectively. The significant distributions of the TAWSS are emphasized (the segments in the solid frames are magnified and labeled with solid star, the dashed frames correspond to the hollow star) (Unit of TAWSS: Pa)

Time average wall shear stress (TAWSS) was evaluated in the stenotic branches, and their variations along the branches are illustrated in Fig. 6. In the figure, H1, H2, H3, H4 indicate high TAWSS, and the L1, L2, L3, L4 indicate low TAWSS for each case. Significant high TAWSS was found in the stenosis area in cases 2, 3 and 4, except for case 1. High TAWSS is also found to be distributed along the branches, as for instance, where the curvature was found in case 1 (labeled with a solid star in H1) and the daughter branches proximal to the bifurcations (labeled with a hollow star in H4).

On the other hand, significant low TAWSS was seen to be located at various sites, including immediate downstream of the stenosis (labeled with a solid star in L1 for case 1), immediate upstream of the stenosis (labeled with a solid star in L2 for case 2) and at the area of the bifurcations (labeled with a solid star in L1 for case 1, a hollow star in L3 for case 3, and a solid star in L4 for case 4).

The maximum TAWSS at the stenosis was 22.07, 57.14, 8.69, 11.6 Pa for cases 1, 2, 3 and 4, respectively; minimum TAWSS at the significant low TAWSS area was 1.2, 1.5, 1.4 and 1.21 Pa for cases 1, 2, 3, and 4, respectively.

### Outcomes of the actual vs. simulated interventions performed for case 3

**Fig. 7:**
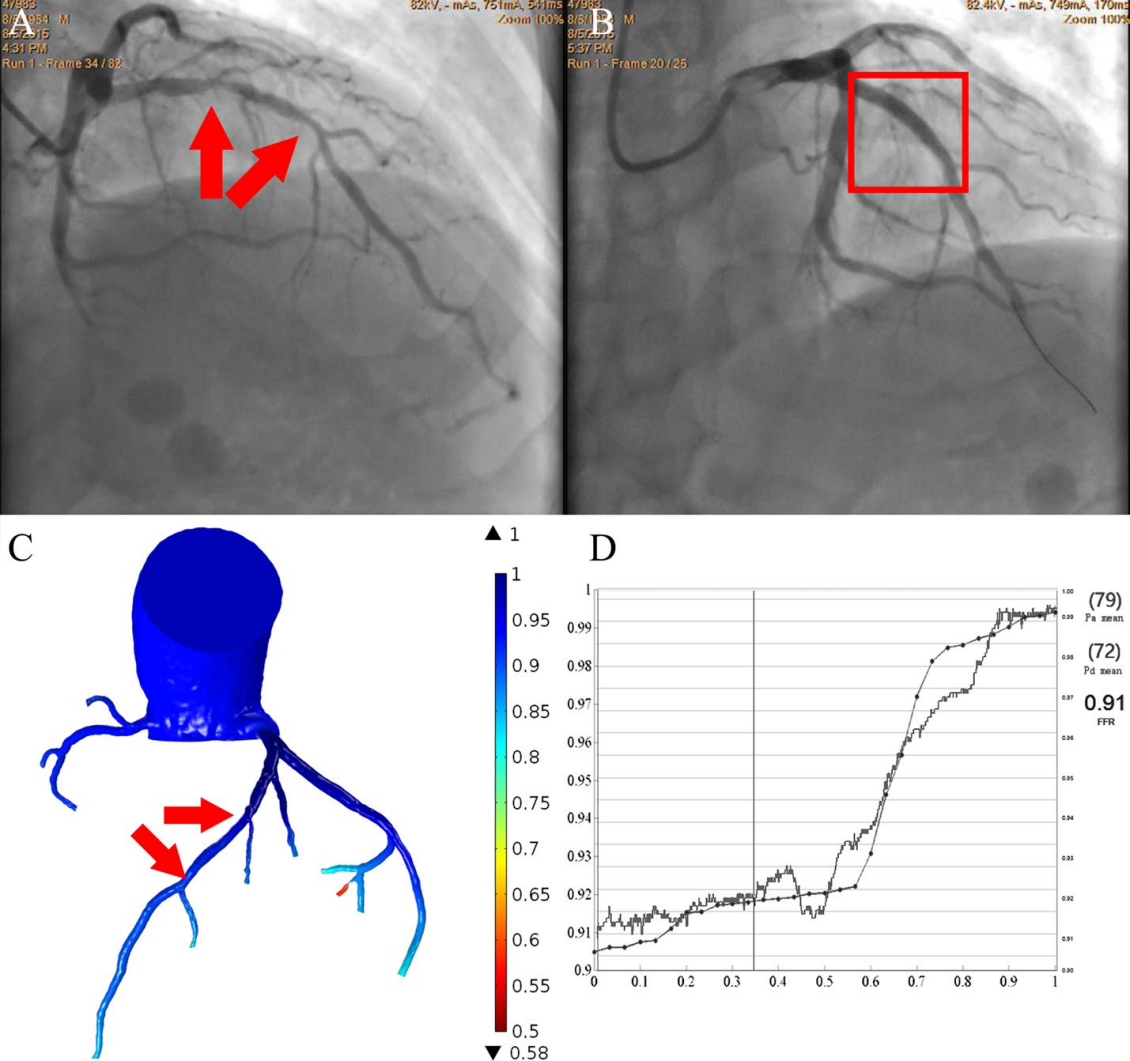
The evaluation of the virtual revascularization for case 3. For the significant bifurcation stenoses (shown in **A**) for case 3, revascularization procedure was performed by placing a stent of length equal to the arterial segment between bifurcations, to restore the cross-section area of the lumen (**B**). Virtual revascularization was then implemented and the FFRCT gradient was determined as shown in **C**. The overlapping of the FFRCT pullback curve (solid line with circle markers) with the measurement (solid line) can be seen in **D**

Treatment had been performed in the stenosis of hemodynamic significance for case 3 (Fig. 7A, B). For the serial stenoses (in case 3, a stent of length equals to the arterial segment between bifurcations was placed to restore the cross-section area of the lumen. Invasive FFR was measured immediately following the PCI procedure and the FFR value along the vessel from the distal stenosis to the ostium of the LAD was recorded (Additional file 1).

Virtual revascularization by virtual stenting was then performed in the image-based reconstruction of the stenoses (Fig. 7C). For the virtual revascularization, the stenotic lumen was repaired, and the cross-section areas of the repaired lumen equaled to the average of the cross-section areas of the immediate upstream and downstream of the stenosis. The pullback curve was then calculated, as shown in Fig. 7. The FFRCT pullback curve was overlapped to the measurement curve as shown in Fig. 7D. The pattern of the FFRCT pullback curve shows good agreement with the FFR (the spearmen correlation factor was over 0.93 with p value < 0.01).

## Discussion

### The geometric specifications

The typical stenotic characteristics in the present cohort were (i) isolated mild stenosis in the middle of LAD, (ii) multiple stenoses at the bifurcation with distal daughter branch of LAD, (iii) multiple stenoses at the adjacent bifurcation of LAD, and (iv) isolated significant stenosis in the middle of RCA. The development of the atherosclerotic plaque can result in different symptoms among individuals during the progression of the disease. Variation of the plaque distribution and the severity of the stenosis are critical factors in the decision-making of the treatment approaches and medical managements. In the present study, patients with suspected significant stenosis based on the clinical symptoms and CTA screening had been performed invasive FFR for diagnostic purpose.

However, current attentions have mainly concentrated on the FFR value distal to the stenosis that neither CTA reconstruction nor FFR could help identifying the culprit stenosis. It is necessary to consider the arterial segment proximal and distal to the stenosis as an integrity, to provide comprehensive information for the intervention planning, especially for the serial stenoses [7]. We had performed the CFD analysis on the patient specific coronary artery trees to determine their global hemodynamic features.

### Effectiveness of the hemodynamic analysis and the pullback curve in relation to the structural variation determination

Hemodynamic analysis is able to provide promising non-invasive functional assessment of the stenosis in previous studies [34]. In this study, we had shown good agreement between FFRCT pullback curve to the invasive FFR measurement. In addition to the FFRCT values distal to the stenoses, the FFRCT pullback curves along the stenotic vessels were determined and compared with the FFR measurement. The study of Tanaka et al. [35] had demonstrated the availability of the image-based FFRCT pullback curve in evaluating the serial stenosis in a single branch. We have further advanced the implementation of the FFRCT pullback curve based vascular geometric evaluations, by comparing the FFRCT pullback curves with the distribution of the target stenotic coronary segment. Our results have shown that the pressure recovery rate was highly correlated to the specific geometric characteristics, including the distribution of the stenosis and the severity of the stenosis. As shown in Figs. 3 and 4, the pressure recovery rate was significantly different between isolated stenosis (case 1) and serial stenoses in a single vessel (case 2 and case 3), regardless of the distribution of the stenosis. The pressure along the stenotic artery recovered immediately over the isolated stenosis, but the pressure recovery over the distal stenosis was limited by the proximal stenosis in the artery with serial stenosis (Additional file 2).

The pullback curves are influenced by the severity of the stenosis in the isolated stenosis and serial stenoses. The distance between the serial stenoses could also be derived from the pullback curves [36] along with the area of hemodynamics significance affected by the stenosis. In our study, the radius of the stenotic artery was found strong correlated with FFRCT and the variation of the radius are significantly affected the variation rate of the FFRCT along the artery. Although the Bland–Altman test (as showed in Fig. 3) demonstrated small deviations between the calculations and the measurements along the culprit vessels. These deviations can mainly be attributed to the images, based on which the coronary arterial models were reconstructed. The deviations were found to be concentrated in the normal segments proximal to the stenosis in cases 1 and 2, while being distributed along the segment between the serial bifurcation stenoses in case 3 and the narrowing segment of the lumen proximal to the stenosis in case 4. Good correlation was achieved between the FFRCT pullback curve and the FFR pullback curve and the pressure gradient values were in good agreement to the FFR values in the functional assessment of significant stenosis. Therefore, the FFRCT pullback curve could have provided more information about the coronary arterial stenosis characteristics compared to the functional assessment of the stenosis alone.

The pull back of the pressure wire during FFR evaluation was performed manually that the delicate work required precise control on the strength and speed in case of emergence complications. But, divergence could be found to the pullback curve of FFR during the procedure because of the pullback speed may be affected by the skill and experience of the operator. The quantification of the distance between serial stenoses was therefore difficult. On the other hand, the FFRCT pullback curve along the vessel could provide accurate distance information between serial stenoses, based on which the interaction factor and culprit stenosis could be further determined.

Finally, the outcome of the virtual revascularization has shown the potential value of more precision PCI being carried out, as well as in predicting the outcome of PCI in serial stenoses guided by FFRCT pullback curve. Detail of the fluctuations found in the measurement were missing in the calculation. The factors that contributed to the fluctuations, however, required further investigation, in order to determine the potential impacts on the design of the PCI procedure and its outcome [37].

In addition to the functional assessment based on the pressure evaluation, hemodynamics analysis could provide significant supplementary information of the geometric characteristics of the stenotic coronary trees. According to Chen et al. [36], the distribution of the serial stenosis was related to the area of the atherosclerosis-prone zone. Similar results were found in our study; in case 2, the long-distance serial stenoses distributed in the parent vessel and the child vessel had limited interaction; in case 3 the serial stenosis located at the adjacent bifurcation contributed to the low WSS area in between the stenosis. On the other hand, by comparing the WSS distribution to the FFR pullback curve, the distribution of high WSS was found over the proximal bifurcation that corresponded to the fluctuation of the FFR from 0 to 0.25 normalized length in Case 1, while the increase of WSS proximal to the stenosis was found to correspond to the reduction of the pressure recovery rate at the proximal segment of RCA. Therefore, the WSS distribution analysis along with the FFRCT pullback curve could provide additional details to the sensitivity of the non-linear structural variations other than just the significant stenosis.

## Limitations

The summation effect of serial stenosis was found in this study, that the FFRCT pullback curve of the serial stenoses presented a reduction of the pressure recovery rate between the stenoses, compared to that of the isolated stenosis. However, the effect of the interactions between the serial coronary stenoses on the hemodynamic parametric distributions do require further investigation, along with the contribution of the specific geometric characteristics such as the bifurcation angle and the curvature of the arteries.

On the other hand, assumptions in the CFD simulation could be limited as the pulsatile patterns along the coronary arteries attenuated from the aorta to arterioles [38]. The 3-dimensional arterial structure concerned in the present study with a minimum diameter over 1 mm, of which the pulsatile nature was preserved. Therefore, the assumptions were appropriate and facilitate the effectiveness of calculations.

In the present study, the subject sample was limited by the condition of multiple isolated stenoses instead of a combination of diffusive stenoses. Although the sample size was limited in the present study, we aim to establish the potential application of the non-invasive functional assessment of coronary vessels with complex stenosis distributions, by involving a larger study sample for more accurate analysis.

## Conclusion

FFR guided PCI surgery has significantly improved the clinical outcome in patients who suffer from ischemic symptoms due to the coronary stenosis. We have evaluated the pressure variation along the stenotic coronary artery based on FFRCT pullback curve, to provide a more comprehensive understanding of the influence of the coronary geometric characteristics on the pressure distribution within it. Our results showed that the combination of FFRCT pullback curve and WSS distributions can provide more accurate evaluation of the serial coronary stenoses. Our study has demonstrated the diagnostic accuracy of FFRCT pullback curve providing noninvasive quantification of the hemodynamics of stenotic coronary arteries with serial lesions, in comparison with the gold standard invasive FFR, to provide a reliable physiological assessment of significant amount of coronary artery disease. Further, we were even able to demonstrate the potential of carrying out virtual revascularization, to enable more precise PCI procedures and improve their outcomes.

## New and noteworthy

The single value of the pressure gradient distal to stenosis could provide limited information about the serial stenoses. We tried to evaluate the FFRCT pullback curve for more comprehensive understanding of the complex distribution of stenosis based on computational fluid dynamics analysis. In combination with wall shear stress, the FFRCT pullback curve analysis could identify the distance between serial stenoses, moreover, the insignificant lumen narrowing with noted increasing of wall shear stress.

## Additional files

**Additional file 1.** CTA image data (part 1).

**Additional file 2.** CTA image data (part 2).

## Abbreviations

CAD: coronary artery disease
FFRF: ractional flow reserve
CTA: computed tomography angiography
FFRCT: computed FFR based on CTA images
MRA: magnetic resonance imaging angiography
ICA: invasive coronary angiography
CFD: computational fluid dynamics
LM: left main coronary artery
LAD: left anterior descending branch
LCX: left circumflex branch
RCA: right coronary artery
